# 3-Heptylidene-4,6-Dimethoxy-3*H*-Isobenzofuran-1-One Is Genotoxic, Increases the Frequency of Cell Death, and Potentiates the Effects of Cyclophosphamide and Cisplatin

**DOI:** 10.3390/molecules28031044

**Published:** 2023-01-20

**Authors:** Silvia Cordeiro das Neves, Flavio Henrique de Araújo, Willian Ayala Correa, Allana Cristina Faustino Martins, Henrique Rodrigues Scherer Coelho, Marcelo Luiz Brandão Vilela, Valter Aragão do Nascimento, Candida Aparecida Leite Kassuya, Dênis Pires de Lima, Adilson Beatriz, Rodrigo Juliano Oliveira, Roberto da Silva Gomes

**Affiliations:** 1 Stem Cell, Cell Therapy and Toxicological Genetics Research Centre (CeTroGen), Medical School, Federal University of Mato Grosso do Sul, Campo Grande 79080-190, MS, Brazil; 2 Graduate Programme in Health and Development in the Midwest Region, Medical School, Federal University of Mato Grosso do Sul, Campo Grande 79070-900, MS, Brazil; 3 Institute of Chemistry, Federal University of Mato Grosso do Sul, Campo Grande 79070-900, MS, Brazil; 4 Department of Pharmaceutical Sciences, North Dakota State University, Fargo, ND 58102, USA; 5 Medical School, Federal University of Mato Grosso do Sul, Campo Grande 79070-900, MS, Brazil; 6 School of Health Sciences, Federal University of Grande Dourados, Dourados 79070-900, MS, Brazil

**Keywords:** Phthalide **1**, in vivo, toxicology, chromosomal damage

## Abstract

3-heptylidene-4,6-dimethoxy-3*H*-isobenzofuran-1-one (Phthalide **1**) is the precursor of three resorcinol lipids that have been described as potential chemotherapeutic agents and capable of potentiating the effects of cyclophosphamide. In this study, we evaluated the genotoxic potential, cell-killing potential, and interactions with cyclophosphamide and cisplatin of phthalide **1**. Twelve groups were created from 120 mice: Negative Control, cyclophosphamide (100 mg/kg), cisplatin (6 mg/kg), Phthalide **1** (5, 10 and 20 mg/kg), and associations of **1** with cyclophosphamide and **1** with cisplatin. The results demonstrate that **1** increases (*p* < 0.05) the frequency of chromosomal damage, liver and kidney cell death, and splenic phagocytosis. The association of **1** with cyclophosphamide and cisplatin demonstrated a chemopreventive effect and, therefore, a reduction (*p* < 0.05) in the frequency of chromosomal damage. However, cell death and splenic phagocytosis did not suffer significant variations. As a result of the above, **1** has potential chemotherapeutic application and may be a candidate for developing a new generation of chemotherapeutics. In addition, it has characteristics to be used as a chemotherapy adjuvant in association with cyclophosphamide and cisplatin since it increases the frequency of cell death induced by chemotherapy. We also reported that the chemopreventive effect of **1**, in association with cyclophosphamide and cisplatin, can prevent adverse effects (induction of DNA damage in non-tumor cells) without interfering with the mode of action of chemotherapy drugs and, therefore, without reducing the induction of cell death.

## 1. Introduction

Resorcinolic lipids, such as cytosporones and their precursors, can interact with the phospholipid bilayers [1], help in the formation of liposomes [2], promote protection against oxidative stress [3], and inhibit bacterial growth [1,2] and tumor cells [4]. In addition, the resorcinol lipid cytosporone B induced apoptosis in tumor cells by interacting with the nuclear orphan receptor Nur77 [2,4]. This fact led different research groups to dedicate themselves to describing the possible chemotherapeutic effects of precursors and cytosporones.

**Figure 1:**
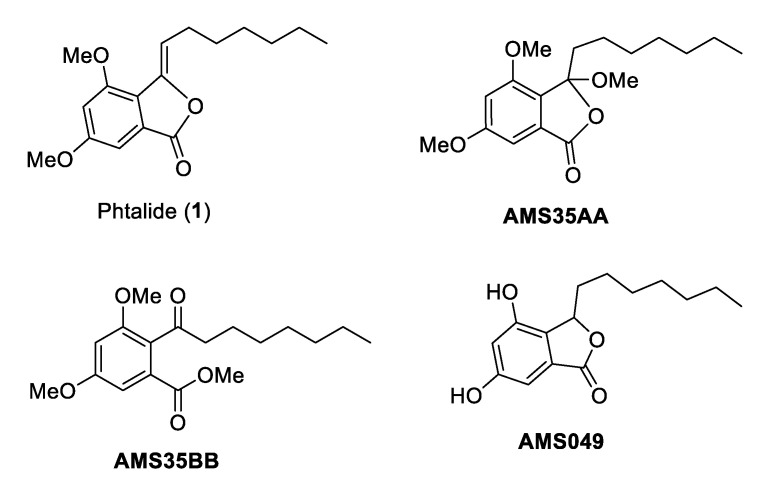
Chemical structures of cytosporone analogs.

We identified a precursor to synthetic cytosporone called 3-heptyl-3,4,6-trimethoxy-3*H*-isobenzofuran-1-one (AMS35AA, Figure 1) as a potential chemotherapeutic agent. AMS35AA showed the ability to increase the frequency of cell death in liver and kidney nodes while promoting the activation of phagocytosis in the spleen, even in the absence of the induction of genotoxic damage. This fact also allowed us to suggest that AMS35AA may be the prototype of more selective chemotherapy since it does not induce DNA damage [5]. Navarro et al. [2] reported the anticancer effect of this compound in a B16F10-induced melanoma model in BC57BL/6 mice. This study demonstrated a reduction of up to 4.59× in tumor weight [2].

The literature has also described the effects of cytosporone 3-heptyl-4,6-dihydroxy-3*H*-isobenzofuran-1-one (AMS049, Figure 1) [6]. The authors suggested that AMS049 is also a candidate for chemotherapy development. However, unlike AMS35AA, this compound induced genomic damage and increased comet frequency. Nevertheless, it maintained a good ability to induce cell death in the liver and kidneys [6].

We also demonstrated that 3,5-dimethoxy-2-octanoyl-benzoic acid methyl ester (AMS35BB, Figure 1) is an efficient candidate for developing a chemotherapy drug. AMS35BB also increased the frequency of cell death and caused genomic damage, unlike AMS35AA, which is similar to that described for AMS049.

To identify potential new cancer chemotherapeutic agents and explain the effects of cytosporones and their precursors on DNA, we devoted our research efforts to studying the effect of this class of compounds. Thus, we suggest that AMS35AA, AMS35BB, and AMS049 are good candidates for developing new chemotherapeutic agents. However, despite the structural similarities of these compounds, we did not observe a pattern in the biological responses. We also did not identify structural differences that could explain variations in biological responses.

To produce these three compounds, we started with the commercially available 3,5-dimethoxybenzoic acid, and by one-step acetylation, **1** was achieved (Figure 1). A methoxylation process created AMS35AA and AMS35BB, and via subsequent hydrogenation followed by demethylation, AMS049 was obtained. Thus, to elucidate the biological responses already described for these three compounds, we chose to evaluate **1** (3-heptylidene-4,6-dimethoxy-3*H*-isobenzofuran-1-one), which is the common precursor. This study aimed to assess the genotoxic effects and the capacity to cause cell death of **1** alone or in association with cyclophosphamide and cisplatin.

The present research aimed to assess the genotoxic effects and ability to induce cell death of compound **1** and its effects in association with cyclophosphamide and cisplatin.

## 2. Results

**Figure 2:**
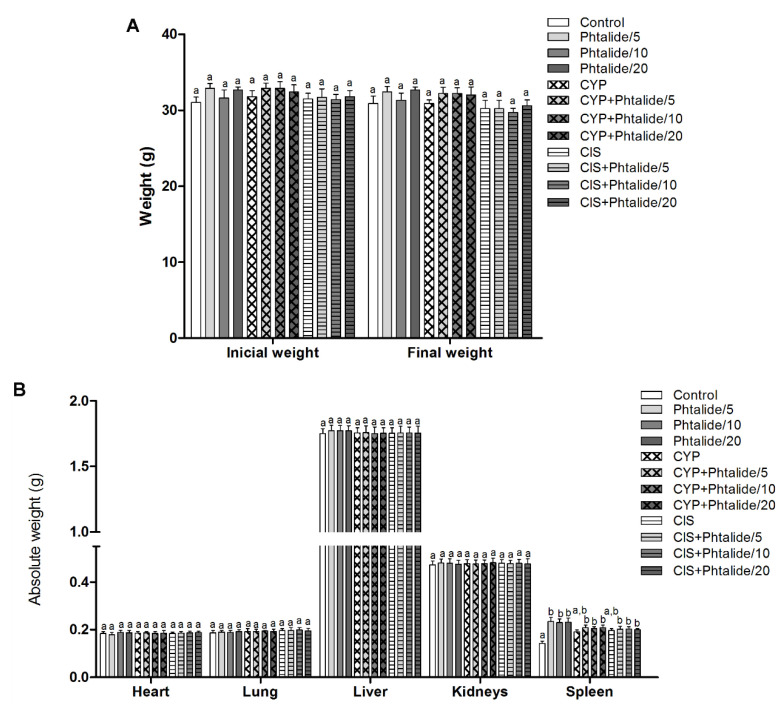
Biometric parameters of mice treated with Phthalide **1**. (**A**) Initial and final weight; (**B**) Absolute organ weight: Heart, lung, kidneys, liver, and spleen. Legend: g: Gram; CYP: Cyclophosphamide; CIS: Cisplatin; Phthalide **1**: 3-heptylidene-4,6-dimethoxy-3*H*-isobenzofuran-1-one; Phthalide **1**/5–5 mg/kg; Phthalide **1**/10–10 mg/kg; Phthalide **1**/20–20 mg/kg. Results are presented as mean ± SEM. Different letters indicate statistically significant differences (Statistical test: ANOVA/Tukey, *p* < 0.05).

The experimental animals started the experiment with similar weights (*p* > 0.05), maintained in the final weight. The mean initial weight ranged from 31.00 ± 0.75 to 32.90 ± 0.63 and the final weight from 29.70 ± 0.57 to 32.70 ± 0.36 (Figure 2A, Table 1).

The organ weights’ evaluation showed no significant differences (*p* > 0.05) between the experimental groups for heart, lung, liver, and kidneys. However, there was an increase (*p* < 0.05) in spleen weight in all groups receiving **1** regardless of the dose (Figure 2B, Table 2).

**Figure 3:**
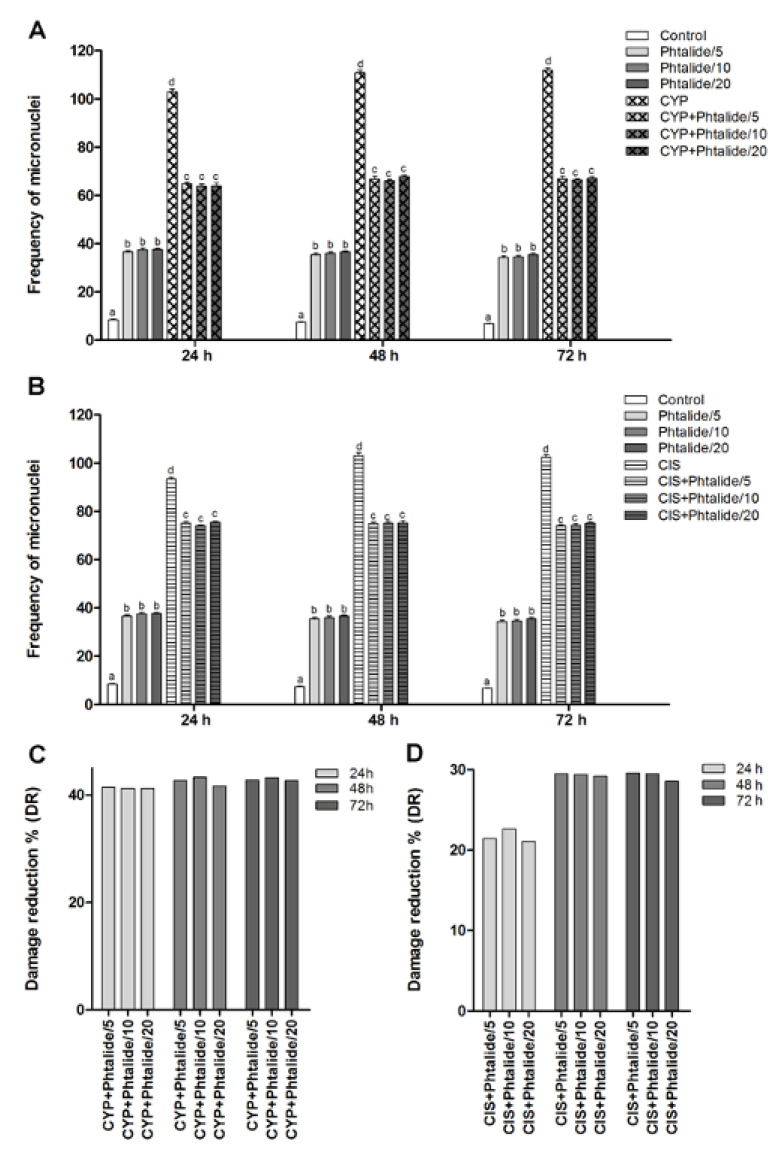
Frequency of micronuclei and percentage of damage reduction in different experimental groups. (**A**) Genotoxicity assessment: Chromosomal lesions in the compounds tested with **1** and **1** associated with cyclophosphamide; (**B**) Genotoxicity assessment: Chromosomal lesions in the compounds tested with **1** and **1** associated with cisplatin; (**C**) Percentage of damage reduction of **1** associated with cyclophosphamide; (**D**) Percentage of **1** damage reduction associated with cisplatin. Legend: g: Gram; CYP: Cyclophosphamide; CIS: Cisplatin; Phthalide **1**: 3-heptylidene-4,6-dimethoxy-3*H*-isobenzofuran-1-one; **1**/5–5 mg/Kg; **1**/10–10 mg/Kg; **1**/20–20 mg/Kg. Results are presented as mean ± SEM. Different letters indicate statistically significant differences (Statistical test: ANOVA/Tukey, *p* < 0.05).

The micronucleus assay demonstrated that cyclophosphamide and cisplatin were efficient in causing DNA damage, and the frequency of injured cells increased by 12.26–16.44× and 11.11–15.06× for cyclophosphamide and cisplatin, respectively (Figure 3A,B, Table 3).

Phthalide **1** was genotoxic at all doses tested (*p* < 0.05). The 5 mg/kg dose caused an increase in the micronucleus frequency by 4.36×, 4.72×, and 5.04× for 24, 48, and 72 h, respectively. The dose of 10 mg/kg increased the micronuclei frequency by 4.46×, 4.79×, and 5.09× for 24, 48, and 72 h, respectively. The dose of 20 mg/kg caused an increase of 4.47×, 4.88×, and 5.21× for the same times, respectively (Figure 3A, Table 3).

The association of **1** with cyclophosphamide demonstrated a chemopreventive effect since it reduced (*p* < 0.05) the frequency of DNA damage (Figure 3A). Damage reduction percentages ranged from 41.22 to 43.27%. There were no significant differences between the tested doses. (Figure 3C).

The association of **1** and cisplatin also demonstrated a chemopreventive effect and, therefore, a significant reduction (*p* < 0.05) in the frequency of DNA damage (Figure 3B). Damage reduction percentages ranged from 21.08 to 29.50% (Figure 3D).

**Figure 4:**
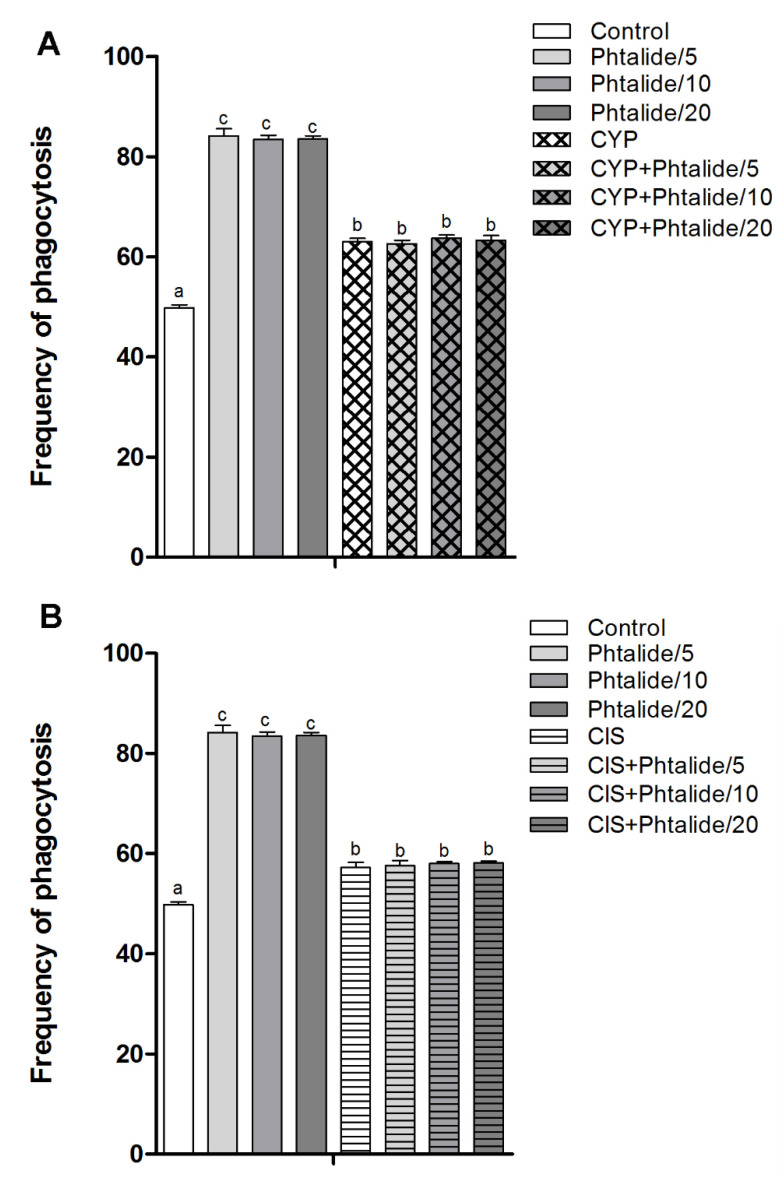
Frequency of splenic phagocytosis. (**A**) Splenic phagocytosis in groups treated with **1** and **1** associated with cyclophosphamide; (**B**) splenic phagocytosis in groups treated with **1** and **1** associated with cisplatin. Legend: g: Gram; CYP: Cyclophosphamide; CIS: Cisplatin; **1**: 3-heptylidene-4,6-dimethoxy-3*H*-isobenzofuran-1-one; **1**/5–5 mg/kg; **1**/10–10 mg/kg; **1**/20–20 mg/kg. Results are presented as mean ± SEM. Different letters indicate statistically significant differences (Statistical test: ANOVA/Tukey, *p* < 0.05).

The splenic phagocytosis assay indicated that cyclophosphamide and cisplatin increased (*p* < 0.05) splendor phagocytosis by 1.26× and 1.15×, respectively (Figure 4A,B).

Phthalide **1** also increased the frequency of phagocytosis by 1.69×, 1.68×, and 1.68× at doses of 5, 10, and 20 mg/kg (Figure 4A, Table 4).

The association of **1** with cyclophosphamide and cisplatin did not reduce the frequency of phagocytosis, which is commonly induced by these chemotherapy agents (Figure 4A,B).

**Figure 5:**
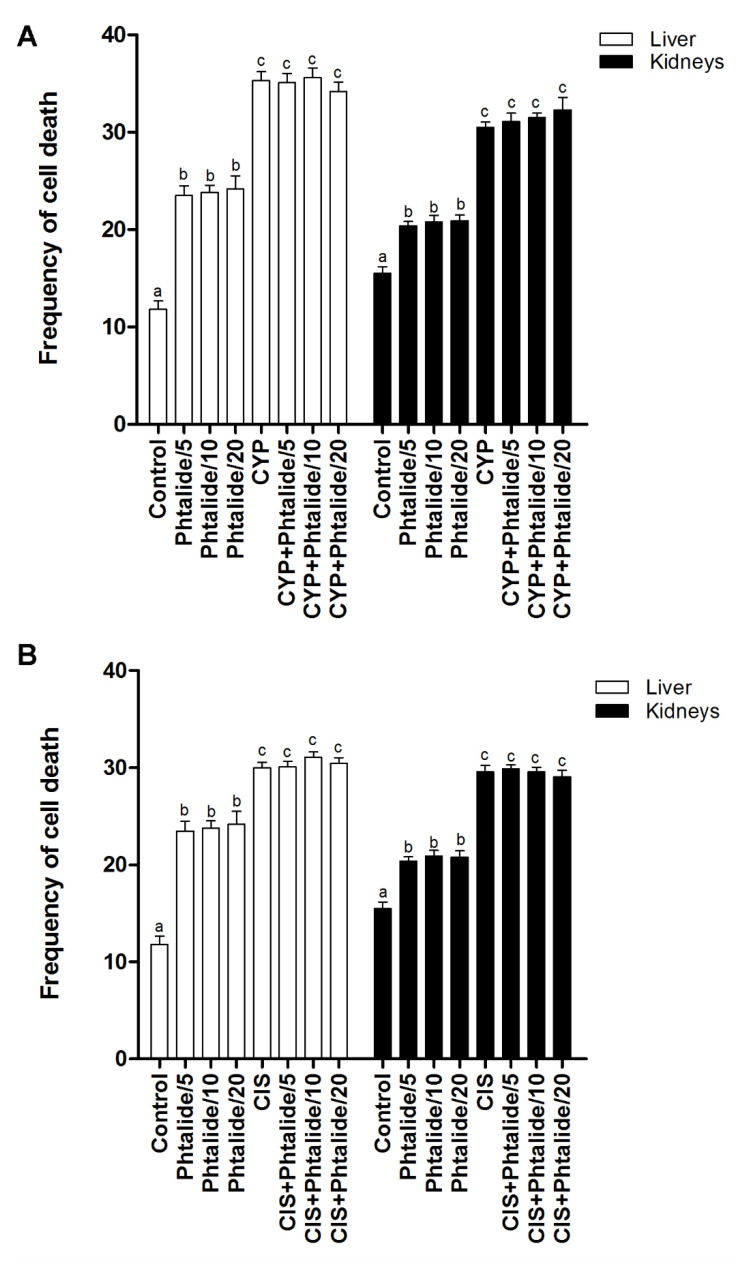
Frequency of cell death. (**A**) Evaluation of cell death in the groups treated with **1** and **1** associated with cyclophosphamide; (**B**) evaluation of cell death in groups treated with **1** and **1** associated with cisplatin. Legend: g: Gram; CYP: Cyclophosphamide; CIS: Cisplatin; **1**: 3-heptylidene-4,6-dimethoxy-3*H*-sobenzofuran-1-one; **1**/5–5 mg/kg; **1**/10–10 mg/kg; **1**/20–20 mg/kg. Results are presented as mean ± SEM. Different letters indicate statistically significant differences (Statistical test: ANOVA/Tukey, *p* < 0.05).

The cell death assay indicated that cyclophosphamide and cisplatin increased (*p* < 0.05) the frequency of cell death in the liver and kidney. Cyclophosphamide increased the frequency in the liver and kidney by 2.99× and 1.97×, respectively (Figure 5A). Cisplatin increased by 2.54× and 1.91× for the same organs, respectively (Figure 5B).

Phthalide **1** increased (*p* < 0.05) the cell death frequency in the liver and kidney. The increase in the liver was 1.99×, 2.02×, and 2.05× for 5, 10, and 20 mg/kg doses, respectively (Figure 3A). In the kidneys, the increases were 13.2×, 1.34×, and 1.35× for the same doses, respectively (Figure 5A, Table 5).

The association of **1** with cyclophosphamide and cisplatin did not reduce (*p* > 0.05) the frequency of cell death in the liver and kidneys induced by these chemotherapy agents (Figure 5A,B, Table 5).

### Molecular Modeling

**Figure 6:**
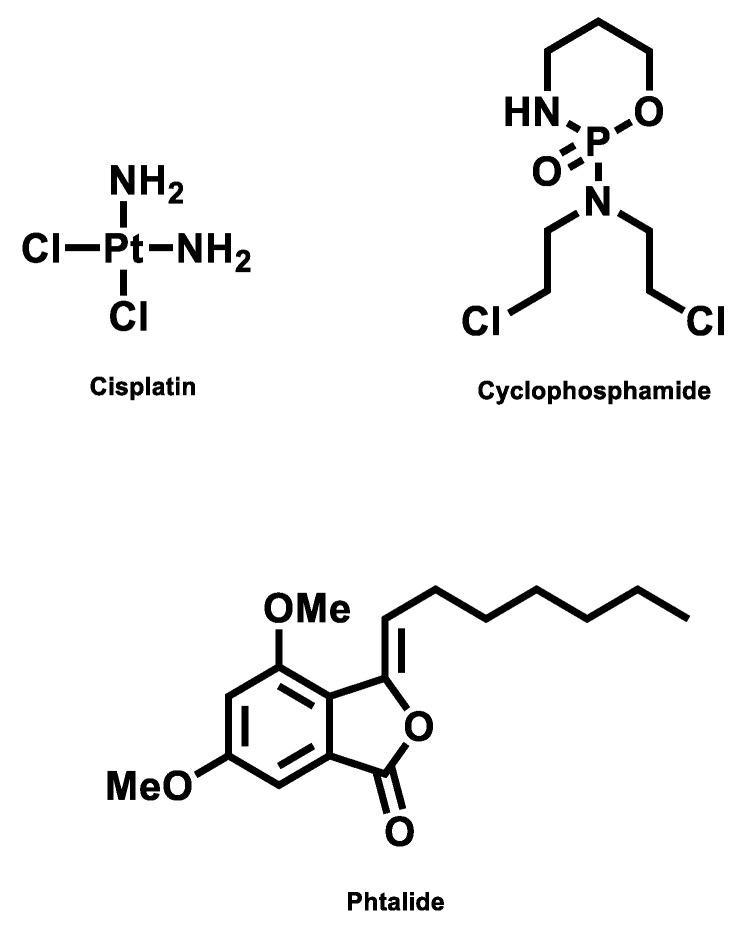
Structure of compounds.

**Figure 7:**
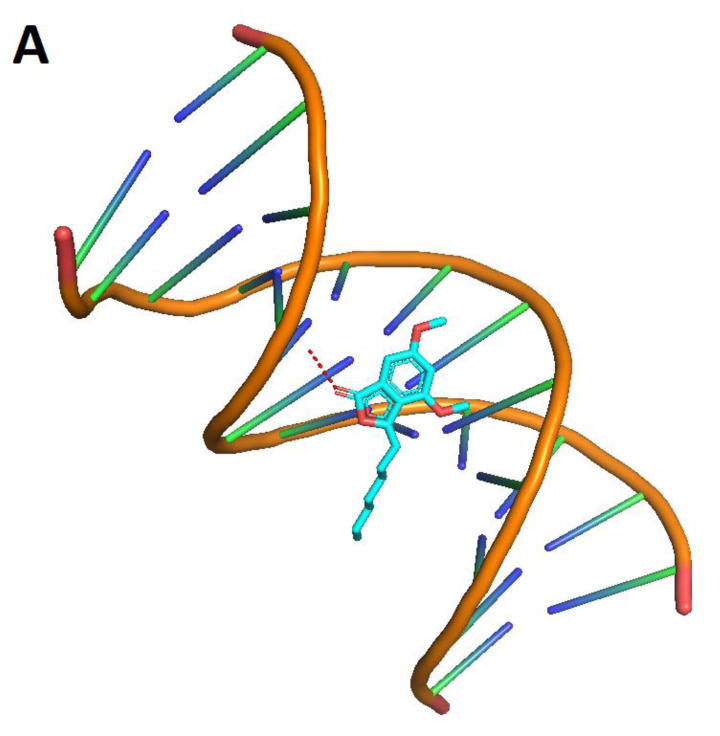

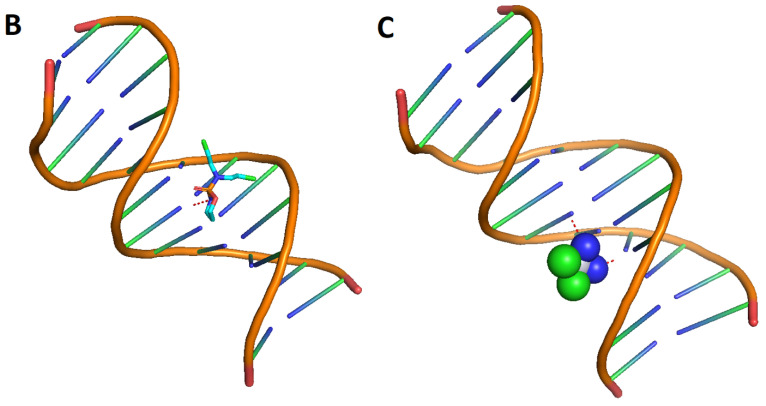
Interactions between the compounds (red dots) and B-DNA: **1** (**A**), cyclophosphamide (**B**), and cisplatin (**C**).

To explore the most feasible binding site, interaction mode and binding affinity docking studies were performed on compounds **1**, cisplatin, and cyclophosphamide (Figure 6) with B-DNA (PDB ID: 1BNA). As shown in Figure 7, the compounds interact with DNA through interactions at the major groove, showing a high affinity for the A-T-rich region. The resulting relative binding energies of docked **1**, cyclophosphamide, and cisplatin are shown in Table 6.

The interactions between the B-DNA and the compounds are shown in Figure 7. In addition, the results indicate a certain hydrogen-bonding interaction between the Phthalide, cyclophosphamide, and cisplatin complexes and DNA.

## 3. Discussion

We described the synthesis of three resorcinol lipids (AMS35AA, AMS35BB, and AMS049) and their effects on short-term biomarkers for carcinogenesis. We also evaluated the effects of these three compounds in association with cyclophosphamide. We observed that all three lipids have important characteristics for developing new chemotherapeutics and/or chemotherapeutic adjuvants. Overall, we also demonstrated that these compounds could enhance the chemotherapeutic effects of cyclophosphamide either by increasing the frequency of DNA damage and cell death or by reducing adverse effects by increasing lymphocyte counts, for example [2,5,6]. However, when analyzing the biological responses of these three compounds, we did not observe a pattern, nor were we able to correlate the different responses to changes in the molecules. Thus, we chose to evaluate **1**, a common precursor of resorcinol lipids, in search of more information about its mechanisms of action.

Phthalide **1** did not induce changes in final weight and organ weight, except for spleen weight. Additionally, no clinical manifestations of toxicity were seen, such as dryness of the mucosa, opacity and bristling of the hair, behavioral abnormalities, skin lesions, lethargy, alterations in walking (locomotor hypoactivity), tremors, decreased food and water intake, and eventually death [7]. Thus, it is suggested that **1** did not induce toxicity. The observed enlargement of the spleen only in the groups treated with **1**, alone or in association with the chemotherapy drug cyclophosphamide, can be explained by the increase in splenic phagocytosis, which was mainly observed in the group treated with Phthalide **1** alone. The three resorcinol lipids that were produced from **1** also showed no signs of toxicity [2,5,6].

Regarding genotoxicity, it was observed that **1** induced an increase in the frequency of chromosomal damage by up to 5.21×. However, this same ability was not observed for the resorptive lipids derived from it, AMS35AA, AMS35BB, and AMS049. These compounds did not cause chromosomal damage when using the same experimental model that was the Swiss mouse [5,6]. AMS35AA also did not induce chromosomal damage in C57BL/6 [2]. However, AMS35BB and AMS049 caused genomic damage [6]. Both genomic damage (assessed by the comet assay) and chromosomal damage (assessed by the micronucleus assay, for example) can be classified as genotoxic damage [8,9]. Genomic damage can be repaired, and chromosomal is already fixed in the cellular genome [10]. Notably, genomic damage can evolve into chromosomal damage [11]. It is considered that **1** causes genetic damage that is more severe than those observed for AMS35BB and AMS049.

In addition to inducing chromosomal damage, **1** also induced increased splenic phagocytosis. This finding was already expected since the spleen can remove micronucleated cells from circulation by activating splenic phagocytosis. Thus, phagocytosis can occur in response to DNA damage as a cellular defense mechanism against genotoxic agents [5,12,13,14,15]. AMS35AA also increased splenic phagocytosis. However, this compound did not increase the frequency of genotoxic damage [5]. It is not uncommon for this type of biological response to occur. According to Oliveira et al. [14], a phagocytosis increase was also registered for IR-01, even in the absence of genotoxic damage.

Phthalide **1** increased the frequency of cell death in both the liver and kidneys. This fact was also observed for the resorcinol lipids, AMS35AA, AMS35BB, and AMS049, derived from **1**. This fact allowed the authors to suggest a good antitumor effect for such compounds [5,6]. Furthermore, the antitumor effect has already been confirmed, for example, for AMS35AA in a solid tumor model induced by B16F10 [2].

A fact that drew attention was that **1** increased cell death in the presence of chromosomal damage. AMS35AA, on the other hand, increased cell death in the absence of genotoxic damage, while AMS35BB and AMS049 increased the frequency of cell death in the presence of genomic and non-chromosomal damage. These facts suggest that AMS35AA could be a more selective chemotherapy prototype because it induces cell death without causing DNA damage. This effect is an important feature for a selective antitumor action because, in this way, chemotherapy would no longer have the side effect of inducing damage to the DNA of healthy cells. The induction of DNA damage is a mechanism of action of many chemotherapy drugs, such as cyclophosphamide [16], doxorubicin [17], and cisplatin [18]. However, one of the side effects of these chemotherapy drugs is that they cause DNA damage [11] in all cells, whether they are tumors or not.

Despite the chromosomal damage induced by **1** and the genomic damage caused by AMS35BB and AMS049, these compounds maintain desirable characteristics for developing chemotherapeutic drugs. In this case, it is necessary to develop a cost–benefit ratio since one of the side effects of chemotherapeutic action is in this induction of DNA damage. However, as previously mentioned, several chemotherapeutic drugs on the market already have this mechanism of action/side effect [16,17,18].

The literature indicates that cell death induced by resorcinol lipids may be mediated by the Nur77 nuclear orphan receptor [19,20] or DNA damage [2,4].

AMS35AA showed cytotoxic effects in MCF-7 cells and induced cell death by apoptosis, mediated by DNA damage, which was confirmed by an increase in the frequency of injured cells in the comet test, as well as by an increase in the gene expression of ATR, p21, p53 and GADD45 [4]. According to Navarro et al. [2], AMS35AA is cytotoxic and genotoxic to B16F10 cells. Cell death occurred via apoptosis and was mediated by DNA damage as it increased the frequency of comets and the expression of ATR, p21, p53, and GAD45AA.

The Nur77 receptor (TR3 or NGFI-B), which is also an option for inducing cell death, is a unique transcription factor that belongs to the nuclear orphan receptor superfamily [21] and is capable of regulating proliferation, differentiation, and apoptosis [22,23]. In tumor cells, Nur77 is an oncogenic survival factor that induces apoptosis by activation in the nucleus and migration to the mitochondria. In mitochondria, it interacts with products of the Bcl-2 gene and induces its conformational change, triggering the release of Cytochrome C and, finally, apoptosis [24,25,26].

The molecular docking technique can contribute to rational drug design and mechanistic studies by placing a small molecule in the binding site of the DNA target-specific region, mainly in a non-covalent mode [27]. Molecular docking can rapidly assess the binding affinities and modes between a target substrate, such as DNA and diverse ligands [28].

Intercalation requires a significant deformation owing to the formation of a binding cavity [29,30] in contrast to minor and major groove binding that does not require major conformational changes of the DNA [29]. Most DNA-binding drugs bind at the B-DNA and show a higher affinity for AT-rich sequences [30,31]. The structure with sequences with alternating A and T bases is generally narrow, allowing favorable van der Waals contacts between the drug and the DNA [30,32] in contrast to GC-rich sequences where bulky amino groups of guanine bases affect the groove geometry [30,33]. Studies on drug–DNA binding were performed to elucidate the energetic origins of the binding in terms of intermolecular forces and induced conformational changes and to develop new drug design strategies [34,35].

The DNA major groove offers more specific contacts for establishing hydrogen bonds with the drug, but van der Waals contacts are less favorable owing to groove dimensions [33]. Furthermore, the major groove is often occupied by proteins whose biological activity can be affected by minor groove-binding drugs [33]. In most cases, drug–DNA binding is non-covalent, although covalent bonds may be formed with reactive ligands [29,30].

The most negative binding energy shows a more potent DNA-binding affinity. Thus, the DNA-binding affinities show a more significant interaction with the compounds at the structure’s major groove region.

Cyclophosphamide is a well-known alkylating agent of the nitrogen mustard type. The phosphoramide mustard, an activated form of cyclophosphamide, alkylates and/or binds to DNA. Its cytotoxic effect is primarily due to cross-linking of strands of DNA and RNA and the inhibition of protein synthesis [36,37]. This mechanism also corroborates the docking results, where the cyclophosphamide interacts between the DNA strands and destabilizes the structure.

Interestingly, the cisplatin mechanism of action is that the drug induces its cytotoxic properties through binding to nuclear DNA and subsequent interference with normal transcription, and/or DNA replication mechanisms [38]. This statement corroborates the docking results, where the cisplatin interacts with two different points of the DNA structure.

Phthalide docking results showed greater interaction value with the DNA structure in the major groove, similar to the cyclophosphamide interaction. This compound behavior can move the chemotherapeutics from the major groove, decreasing the interaction stability and reducing the time of interaction between DNA and the chemotherapeutics.

Phthalide **1**, in combination with cyclophosphamide and cisplatin, did not increase the frequency of phagocytosis. This fact was already expected since the association of **1** with chemotherapy agents had a chemopreventive effect, reducing the induction of DNA damage. Regarding this fact, it only reduces the frequency of circulating micronucleated cells and reduces splenic activity. As previously reported, the spleen sequesters cells with DNA damage, for example [5,12,13,14,15].

A similar situation was reported for AMS35BB and AMS049, where these two compounds prevented DNA damage (genomic damage) and chemopreventive action without changing the frequency of phagocytosis induced by cyclophosphamide [6]. Furthermore, despite preventing genomic damage, AMS35AA increased chromosomal damage induced by cyclophosphamide (even though it was not statistically significant) and increased splenic phagocytosis [5].

Phthalide **1** increased the frequency of cell death in association with cyclophosphamide and cisplatin, and it is suggested that this may have happened even in chemopreventive action. Despite **1** reducing DNA damage, there was still an increase in cell death. It is suggested that this fact may have occurred because resorcinol lipids can cause cell death mediated by activation of the orphan nuclear receptor Nur77 as previously reported [19,20]. Action via this route was also suggested for AMS35AA, AMS35BB, and AMS049 [5,6].

Given the above, we consider that **1**, as well as its derived lipids, has characteristics of interest for developing new chemotherapeutics since it increases the frequency of cell death. In addition, it is assumed that its mechanism of action will involve DNA damage since it increases the frequency of micronuclei, an important biomarker for other chemotherapeutic agents already commercialized.

Notably, similar to its derivatives, **1** can potentiate the effects of cyclophosphamide and thus increase the induction of cell death. Therefore, it could be an important chemotherapeutic adjunct. Additionally, its chemopreventive action can protect healthy cells without negatively interfering with the cell-death-inducing effects of cyclophosphamide. On the other hand, even protecting the DNA (chemopreventive action) continues to potentiate cell death induced by cyclophosphamide.

The present study and the comparison of the biological activities of Phthalide **1** with its derivatives did not allow for the elucidation of the different biological responses presented in this study and those already described [5,6]. Thus, we suggest more studies with methylated and non-methylated molecules to allow a structure-activity study.

Moreover, this work contributes unprecedentedly to the literature in the area by elucidating that **1** can also potentiate the chemotherapeutic effects of cisplatin since none of its derivatives have been tested in association with this chemotherapeutic.

## 4. Material and Methods

### 4.1. Chemical Agents, Animals, and Experimental Design

Two positive controls were used: Cyclophosphamide (Fosfaseron^®^, Ítaca Laboratories, REG. M.S. No. 1.2603.0056.002-1; Batch 063020, Campo Grande, Brazil) at a dose of 100 mg/kg body weight (b.w.) administered intraperitoneally (i.p.) [5,39] and cisplatin (Intas Pharmaceuticals Laboratory LTD, REG. M.S.1.5537.0002.003-7; Matoda 382210, Índia) at a dose of 6 mg/kg (b.w.; i.p.) [14,40]. The cyclophosphamide was diluted in Mili-Q water.

Phthalide **1** was readily prepared by treating 3,5-dimethoxybenzoic acid with octanoyl chloride in the presence of AlCl_3_. This compound has been characterized by ^1^H and ^13^C NMR spectroscopy, and the data match those reported in the literature [1].

Phthalide **1** was first dissolved in DMSO (1%) and subsequently diluted in Mili-Q water (final concentration of 1% DMSO) and administered at doses of 5, 10, and 20 mg/kg (b.w.; i.p.) [41].

In this experiment, 120 sexually mature male (approximately 8–10 weeks) Swiss mice (*Mus musculus*) from the Central Animal Facility of the Federal University of Mato Grosso do Sul (UFMS) were divided into 12 experimental groups each (*n* = 10 animals).

The animals were kept in polypropylene boxes covered with litter and fed commercial feed (Nuvital^®^, Campo Grande, Brazil) and filtered water ad libitum. Temperature and luminosity were controlled using a twelve-hour photoperiod (12 h of light: 12 h of darkness), with a temperature of 22 ± 2 °C and humidity of 55% ± 10 in a ventilated shelf (ALESCO®, Campo Grande, Brazil).

The experiment was carried out following the Universal Declaration of Animal Rights guidelines with approval from the Ethics committee on Animal Experimentation (CEUA/UFMS) under protocol #399/2012.

The 12 experimental groups were divided as listed below:

Control group: The animals received **1** vehicle (DMSO 1%) and cyclophosphamide (physiological solution—NaCl 0.9%) at a dose of 0.1 mL/10 g b.w; i.p) simultaneously.

CYP group: The animals received **1** vehicle (0.1 mL/10 g b.w.) and cyclophosphamide (100 mg/kg b.w., i.p.) simultaneously.

CIS group: The animals received **1** vehicle (0.1 mL/10 g p.c.) and cisplatin (6 mg/kg p.c., i.p.) simultaneously.

Phthalide **1** group: The animals received **1**, in three different doses (5 mg/kg; 10 mg/kg, and 20 mg/kg b.w., i.p.) and the cyclophosphamide vehicle (0.1 mL/10 g b.w., i.p.) simultaneously.

CYP + Phtalide group (Phthalide **1** + cyclophosphamide): The animals received 1 (in three different doses—5 mg/kg; 10 mg/kg, and 20 mg/kg b.w., i.p.) and cyclophosphamide (100 mg/kg b.w., i.p.) simultaneously.

CIS + Phtalide group (Phthalide **1** + cisplatin): The animals received **1** (in three different doses—5 mg/kg; 10 mg/kg, and 20 mg/kg b.w., i.p.) and cisplatin (6 mg/kg b.w., i.p.) simultaneously.

### 4.2. Biological Assays

#### 4.2.1. Micronucleus Assay in Peripheral Blood

The micronucleus assay in peripheral blood was performed according to Hayashi et al. [42] with modifications by Oliveira et al. [43]. A 20 µL aliquot of peripheral blood was added to the slide previously coated with 20 μL of Acridine Orange (1.0 mg/mL). Then a cover slip was deposited on the biological material. The slide remained in a freezer (20 °C) for two weeks. The analysis was performed in an epifluorescence microscope (Bioval^®^, Model L 2000 A), using a 400× objective, with a 420–490 nm excitation filter and a 520 nm barrier filter. Two thousand cells/animal were analyzed.

#### 4.2.2. Splenic Phagocytosis Assay

Twenty microliters of Acridine Orange (1.0 mg/mL) were used to cover the surface of a previously heated slide. After 1/3 of the spleen was macerated in saline solution, 100 µL of cell suspension was placed on the stained slide. Then, a coverslip was placed. The slides were stored in a freezer for further analysis. The analysis was performed using a fluorescence microscope (Bioval^®^, Model L 2000 A) at a magnification of 400× with a 420–490 nm filter and a 520 nm barrier filter. One hundred cells per animal were analyzed. The analysis of cells with evidence or absence of phagocytosis was based on the description by Hayashi et al. [42] with modifications by Carvalho et al. [6].

#### 4.2.3. Cell Death Assay

One hundred microliters of liver and/or kidney macerated solution were used to make an extension on a glass side. After this process, the slide was fixed in Carnoy for 10 min, and soon after, it was subjected to different decreasing concentrations of ethanol (95–25%). Then, it was submitted to baths of the McIlvane buffer for 10 min, stained in Acridine Orange (0.01%, 5 min), and washed in McIlvane buffer for 10 min. The identification of cells undergoing apoptosis was performed by analyzing DNA fragmentation patterns [6].

#### 4.2.4. Calculation of Damage Reduction Percentage (DR%)

The damage reduction percentage is used to assess the chemopreventive capacity of a substance when it is associated with a substance that is inducing wide damage. For this evaluation, the formula proposed by [44,45] was used:

(1) $$RD\;or\;ID\left(\%\right)=\left(\frac{Mean\;of\;positive\;control\;-Mean\;of\;associate\;group}{Mean\;of\;positive\;control-Mean\;of\;negative\;control}\right)\times 100$$

The damage reduction percentage was also adapted for phagocytosis and cell death assays.

### 4.3. Statistical Analysis

ANOVA analyzed data with Tukey’s posteriori test using GraphPad Prism Software (version 3.02; GraphPad Software Inc., San Diego, CA, USA). Values were expressed as mean ± SEM and the significance level was *p* < 0.05.

### 4.4. Molecular Docking

#### 4.4.1. Computational Details

The virtual protocol, including molecular docking, was performed on the DELL^®^ Workstation computer, with Intel^®^ Xeon E5-1660 processor, 3.3 GHz, 4 CPUs, NVIDIA^®^ GeForce RTX 2060 graphics card, RAM 8 GB, under the Windows^®^ operating system.

#### 4.4.2. Protein Preparation

The X-Ray diffraction-based crystal structure of a B-DNA Dodecamer (PDB ID: 1BNA) https://doi.org/10.1073/pnas.78.4.2179 (accessed on 10 May 2022) was retrieved from the Protein Data Bank https://www.rcsb.org/ (accessed on 10 May 2022) with a 1.9 Å resolution for the crystal structure of the synthetic DNA dodecamer d (CpGpCpGpApApTpTpCpGpCpG). The water molecules were removed by operating Discovery Studio BIOVIA [46].

#### 4.4.3. Molecular Modeling

The set of 3 compounds was built in the ChemDraw program https://www.perkinelmer.com/product/chemdraw-professional-chemdrawpro (accessed on 10 May 2022) from 2D structures of the series. All geometries were virtually constructed and optimized using molecular mechanics (MM+) and semiempirical Austin Model 1 (AM1) methods using HyperChem 7 program http://www.hyper.com (accessed on 10 August 2022). The output files were converted to input files .mol2 in the Discovery Studio BIOVIA program using Open Babel (http://openbabel.org/wiki/Main_Page) and then converted to .pdb files as input files to HDOCK http://hdock.phys.hust.edu.cn/ (accessed on 10 August 2022).

To predict the best orientation and conformation of the compounds to the BDNA in silico, the HDOCK web server program was used.

#### 4.4.4. Target Selection and Molecular Docking Simulations

The synthetic B-DNA Dodecamer crystallized structure was obtained through the RSCB PDB under code 1BNA. https://www.rcsb.org/structure/1BNA (accessed on 10 May 2022). Then, docking simulations were performed for each inhibitor candidate on the corresponding target. This procedure was performed using the HDOCK webserver [47]. Thus, the Docking score was evaluated.

Before docking using HDOCK, the B-DNA and the compounds were uploaded as .pdb files into the web server. The complexes generated by HDOCK were selected using the best model between the results, which presented the best docking score. The complex structures of the protein site (.pdb) and the ligands (.pdb) were inserted into the BIOVIA Discovery Studio visualizer program to detail the interactions between the ligands and the participating DNA bases.

## 5. Conclusions

In conclusion, **1** has clinical applicability and could be a candidate for developing a new generation of the chemotherapeutic agent. In addition, **1** has characteristics that can be used as a chemotherapy adjuvant in association with cyclophosphamide and cisplatin since **1** increases the frequency of cell death induced by chemotherapy. Regarding the association of these compounds, it was also reported that the chemopreventive effect of **1,** when associated with cyclophosphamide and cisplatin, can prevent adverse effects (the induction of DNA damage in non-tumor cells) without interfering with the mechanism of action of chemotherapy drugs and, therefore, without reducing the induction of cell death.

## Figures and Tables

**Table 1:** Mean ± Standard error of initial and final weight of Swiss mice treated with different doses of **1**.

Experimental Groups	Initial Weight	Final Weight
Control	31.00 ± 0.75 ^a^	30.90 ± 0.97 ^a^
**1** 5 mg/kg	32.90 ± 0.63 ^a^	32.40 ± 0.75 ^a^
**1** 10 mg/kg	31.60 ± 1.10 ^a^	31.30 ± 0.95 ^a^
**1** 20 mg/kg	32.70 ± 0.36 ^a^	32.70 ± 0.36 ^a^
cyclophosphamide (CYP)	31.80 ± 0.82 ^a^	30.90 ± 0.47 ^a^
CYP + **1** 5 mg/kg	32.90 ± 0.68 ^a^	32.20 ± 0.83 ^a^
CYP + **1** 10 mg/kg	32.90 ± 0.89 ^a^	32.20 ± 0.77 ^a^
CYP + **1** 20 mg/kg	32.40 ± 0.95 ^a^	32.00 ± 1.07 ^a^
cisplatin (CIS)	31.50 ± 0.74 ^a^	30.20 ± 0.84 ^a^
CIS + **1** 5 mg/kg	31.70 ± 1.12 ^a^	30.20 ± 1.12 ^a^
CIS + **1** 10 mg/kg	31.40 ± 0.69 ^a^	29.70 ± 0.57 ^a^
CIS + **1** 20 mg/kg	31.80 ± 0.82 ^a^	30.60 ± 0.76 ^a^

Negative control—DMSO 1%; cyclophosphamide—100 mg/kg b.w., i.p.; cisplatin—6 mg/kg, b.w., i.p. **1**—Phthalide **1** at doses 5, 10, 20 mg/kg b.w., i.p. CYP + **1**-cyclophosphamide—100 mg/kg (b.w.; i.p.) + **1** at doses 5, 10, 20 mg/kg (b.w., i.p.); CIS + **1**-cisplatin—6 mg/kg (p.c.; i.p.) + **1** at doses 5, 10, 20 mg/kg (b.w., i.p.). Different letters indicate statistically significant differences. Statistical test: ANOVA/Tukey.

**Table 2:** Mean ± Standard error of the absolute weights of Swiss mice organs collected after the experimental period.

Experimental Groups	Heart	Lung	Liver	Kidneys	Spleen
Control	0.184 ± 0.009 ^a^	0.187 ± 0.009 ^a^	1.749 ± 0.039 ^a^	0.472 ± 0.017 ^a^	0.142 ± 0.010 ^a^
**1** 5 mg/kg	0.179 ± 0.010 ^a^	0.190 ± 0.007 ^a^	1.772 ± 0.042 ^a^	0.480 ± 0.017 ^a^	0.234 ± 0.018 ^b^
**1** 10 mg/kg	0.188 ± 0.010 ^a^	0.189 ± 0.008 ^a^	1.773 ± 0.038 ^a^	0.479 ± 0.019 ^a^	0.230 ± 0.015 ^b^
**1** 20 mg/kg	0.187 ± 0.010 ^a^	0.192 ± 0.009 ^a^	1.772 ± 0.038 ^a^	0.475 ± 0.017 ^a^	0.231 ± 0.018 ^b^
cyclophosphamide (CYP)	0.186 ± 0.007 ^a^	0.192 ± 0.012 ^a^	1.754 ± 0.042 ^a^	0.478 ± 0.017 ^a^	0.191 ± 0.008 ^a,b^
CYP + **1** 5 mg/kg	0.187 ± 0.006 ^a^	0.193 ± 0.009	1.757 ± 0.052 ^a^	0.476 ± 0.017 ^a^	0.208 ± 0.012 ^b^
CYP + **1** 10 mg/kg	0.184 ± 0.008 ^a^	0.195 ± 0.012 ^a^	1.751 ± 0.049 ^a^	0.478 ± 0.015 ^a^	0.204 ± 0.008
CYP + **1** 20 mg/kg	0.186 ± 0.011 ^a^	0.192 ± 0.010 ^a^	1.752 ± 0.050 ^a^	0.481 ± 0.020 ^a^	0.209 ± 0.010 ^b^
cisplatin (CIS)	0.184 ± 0.006 ^a^	0.196 ± 0.009 ^a^	1.752 ± 0.044 ^a^	0.479 ± 0.016 ^a^	0.196 ± 0.008 ^a,b^
CIS + **1** 5 mg/kg	0.186 ± 0.010 ^a^	0.197 ± 0.012 ^a^	1.756 ± 0.051 ^a^	0.477 ± 0.014 ^a^	0.202 ± 0.012 ^b^
CIS + **1** 10mg/kg	0.187 ± 0.007 ^a^	0.199 ± 0.010 ^a^	1.754 ± 0.047 ^a^	0.479 ± 0.017 ^a^	0.203 ± 0.010 ^b^
CIS + **1** 20mg/kg	0.188 ± 0.006 ^a^	0.195 ± 0.011 ^a^	1.753 ± 0.055 ^a^	0.476 ± 0.022 ^a^	0.200 ± 0.005 ^b^

Negative control—DMSO 1%; cyclophosphamide—100 mg/kg b.w., i.p.; cisplatin—6 mg/kg, b.w., ip. **1**-**1** at doses 5, 10, 20 mg/kg b.w., i.p. CYP + **1**-cyclophosphamide—100 mg/kg (b.w.; i.p.) + **1** at doses 5, 10, 20 mg/kg (b.w., i.p.); CIS + **1**-cisplatin—6 mg/kg (p.c.; i.p.) + **1** at doses 5, 10, 20 mg/kg (b.w., i.p.). Different letters indicate statistically significant differences. Statistical test: ANOVA/Tukey.

**Table 3:** Mean frequency ± Standard error of mean and percentage of damage reduction referring to micronucleus assays in peripheral blood of Swiss mice (males).

Experimental Groups	Average Frequency of Micronuclei			Damage Reduction Percentage		
	24 h	48 h	72 h	24 h	48 h	72 h
				Experiment 1		
Control	8.40 ± 0.30 ^a^	7.50 ± 0.27 ^a^	6.80 ± 0.20 ^a^	-	-	-
cyclophosphamide (CYP)	103.00 ± 1.00 ^d^	110.80 ± 0.97 ^d^	111.80 ± 1.08 ^d^	-	-	-
**1** 5 mg/kg	36.60 ± 0.54 ^b^	35.40 ± 0.67 ^b^	34.30 ± 0.67 ^b^	-	-	-
**1** 10 mg/kg	37.50 ± 0.63 ^b^	35.90 ± 0.69 ^b^	34.60 ± 0.50 ^b^	-	-	-
**1** 20 mg/kg	37.60 ± 0.45 ^b^	36.60 ± 0.45 ^b^	35.40 ± 0.67 ^b^	-	-	-
CYP + **1** 5 mg/kg	64.80 ± 0.59 ^c^	66.70 ± 1.29 ^c^	66.90 ± 1.28 ^c^	41.43	42.69	42.76
CYP + **1** 10 mg/kg	63.80 ± 0.78 ^c^	66.10 ± 0.50 ^c^	66.40 ± 0.47 ^c^	41.22	43.27	43.23
CYP + **1** 20 mg/kg	64.00 ± 1.23 ^c^	67.80 ± 0.61 ^c^	67.00 ± 0.80 ^c^	41.22	41.62	42.66
				**Experiment 2**		
Control	8.40 ± 0.30 ^a^	7.50 ± 0.27 ^a^	6.80 ± 0.20 ^a^	-	-	-
cisplatin (CIS)	93.30 ± 0.84 ^d^	103.10 ± 1.24 ^d^	102.40 ± 1.08 ^d^	-	-	-
**1** 5 mg/kg	36.60 ± 0.54 ^b^	35.40 ± 0.67 ^b^	34.30 ± 0.67 ^b^	-	-	-
**1** 10 mg/kg	37.50 ± 0.63 ^b^	35.90 ± 0.69 ^b^	34.60 ± 0.50 ^b^	-	-	-
**1** 20 mg/kg	37.60 ± 0.45 ^b^	36.60 ± 0.45 ^b^	35.40 ± 0.67 ^b^	-	-	-
CIS + **1** 5 mg/kg	75.10 ± 0.70 ^c^	74.90 ± 0.65 ^c^	74.10 ± 0.38 ^c^	21.44	29.50	29.60
CIS + **1** 10 mg/kg	74.10 ± 0.31 ^c^	75.00 ± 0.74 ^c^	74.20 ± 0.61 ^c^	22.61	29.39	29.50
CIS + **1** 20 mg/kg	75.40 ± 0.45 ^c^	75.20 ± 0.94 ^c^	75.10 ± 0.38 ^c^	21.08	29.18	28.56

Negative control—DMSO 1%; cyclophosphamide—100 mg/kg b.w., i.p.; cisplatin—6 mg/kg, b.w., ip. **1**-**1** at doses 5, 10, 20 mg/kg b.w., i.p. CYP + **1**-cyclophosphamide—100 mg/kg (b.w.; i.p.) + **1** at doses 5, 10, 20 mg/kg (b.w., i.p.); CIS + **1**-cisplatin—6 mg/kg (p.c.; i.p.) + **1** at doses 5, 10, 20 mg/kg (b.w., i.p.). Different letters indicate statistically significant differences. Statistical test: ANOVA/Tukey.

**Table 4:** Absolute value, mean ± Standard error, and percentage referring to the splenic phagocytosis assay in Swiss mice.

Experimental Group	Total Cells Analyzed	Total Cells without Evidence of Phagocytosis A.V Mean ± SE Percentage			Total Cells with Evidence of Phagocytosis A.V Mean ± SE Percentage		
Experiment 1 (CYP)							
Control	1000	502	50.20 ± 0.64 ^c^	50.2	498	49.80 ± 0.64 ^a^	49.8
cyclophosphamide (CYP)	1000	370	37.00 ± 0.71 ^b^	37.0	630	63.00 ± 0.71 ^b^	63.0
**1** 5 mg/kg	1000	158	15.80 ± 1.40 ^a^	15.8	842	84.20 ± 1.40 ^c^	84.2
**1** 10 mg/kg	1000	165	16.50 ± 0.80 ^a^	16.5	835	83.50 ± 0.80 ^c^	83.5
**1** 20 mg/kg	1000	164	16.40 ± 0.62 ^a^	16.4	836	83.60 ± 0.62 ^c^	83.6
CYP + **1** 5 mg/kg	1000	374	37.40 ± 0.72 ^b^	37.4	626	62.60 ± 0.72 ^b^	62.6
CYP + **1** 10 mg/kg	1000	363	36.30 ± 0.70 ^b^	36.3	637	63.70 ± 0.70 ^b^	63.7
CYP + **1** 20 mg/kg	1000	367	36.70 ± 0.96 ^b^	36.7	633	63.30 ± 0.96 ^b^	63.3
**Experiment 2 (CIS)**							
Control cisplatin (CIS)	1000	502	50.20 ± 0.64 ^c^	50.2	498	49.80 ± 0.64 ^a^	49.8
	1000	428	42.80 ± 1.00 ^b^	42.8	572	57.20 ± 1.00 ^b^	57.2
**1** 5 mg/kg	1000	158	15.80 ± 1.40 ^a^	15.8	842	84.20 ± 1.40 ^c^	84.2
**1** 10 mg/kg	1000	165	16.50 ± 0.80 ^a^	16.5	835	83.50 ± 0.80 ^c^	83.5
**1** 20 mg/kg	1000	164	16.40 ± 0.62 ^a^	16.4	836	83.60 ± 0.62 ^c^	83.6
CIS + **1** 5 mg/kg	1000	424	42.40 ± 1.01 ^b^	42.4	576	57.60 ± 1.01 ^b^	57.6
CIS + **1** 10 mg/kg	1000	420	42.00 ± 0.40 ^b^	42.0	580	58.00 ± 0.40 ^b^	58.0
CIS + **1** 20 mg/kg	1000	419	41.90 ± 0.38 ^b^	41.9	581	58.10 ± 0.38 ^b^	58.1

Negative control—DMSO 1%; cyclophosphamide—100 mg/kg b.w., i.p.; cisplatin—6 mg/kg, b.w., i.p. **1**-**1** at doses 5, 10, 20 mg/kg b.w., i.p. CYP + **1**-cyclophosphamide—100 mg/kg (b.w.; i.p.) + **1** at doses 5, 10, 20 mg/kg (b.w., i.p.); CIS + **1**-cisplatin—6 mg/kg (p.c.; i.p.) + **1** at doses 5, 10, 20 mg/kg (b.w., i.p.). Legend: A.V.—Absolute value. Different letters indicate statistically significant differences. Statistical test: ANOVA/Tukey.

**Table 5:** Absolute value, mean ± Standard error of liver and kidney cells in apoptosis, Swiss mice (males), treated with different doses of **1**, associated with cyclophosphamide and cisplatin.

Experimental Groups	Total Cells Analyzed	Total Cells in Apoptosis in the Liver A.V Mean ± SE Percentage			Total Cells in Apoptosis in the Kidneys A.V Mean ± SE Percentage		
Experiment 1 (CYP)							
Control	1000	118	11.80 ± 0.88 ^a^	11.8		15.50 ± 0.69 ^a^	15.5
cyclophosphamide (CYP)	1000	353	35.30 ± 0.93 ^c^	35.3		30.50 ± 0.56 ^c^	30.5
**1** 5 mg/kg	1000	235	23.50 ± 0.99 ^b^	23.5		20.40 ± 0.43 ^b^	20.4
**1** 10 mg/kg	1000	238	23.80 ± 0.75 ^b^	23.8		20.80 ± 0.66 ^b^	20.8
**1** 20 mg/kg	1000	242	24.20 ± 1.33 ^b^	24.2		20.90 ± 0.61 ^b^	20.9
CYP + **1** 5 mg/kg	1000	351	35.10 ± 0.91 ^c^	35.1		31.10 ± 0.86 ^c^	31.1
CYP + **1** 10 mg/kg	1000	356	35.60 ± 0.99 ^c^	35.6		31.50 ± 0.49 ^c^	31.5
CYP + **1** 20 mg/kg	1000	342	34.20 ± 0.94 ^c^	34.2		32.30 ± 1.28 ^c^	32.3
**Experiment 2 (CIS)**							
Control cisplatin (CIS)	1000	118	11.80 ± 0.88 ^a^	11.8	155	15.50 ± 0.69 ^a^	15.5
	1000	300	30.00 ± 0.57 ^c^	30.0	296	29.60 ± 0.65 ^c^	29.6
**1** 5 mg/kg	1000	235	23.50 ± 0.99 ^b^	23.5	204	20.40 ± 0.43 ^b^	20.4
**1** 10 mg/kg	1000	238	23.80 ± 0.75 ^b^	23.8	209	20.90 ± 0.61 ^b^	20.9
**1** 20 mg/kg	1000	242	24.20 ± 1.33 ^b^	24.2	208	20.80 ± 0.66 ^b^	20.8
CIS + **1** 5 mg/kg	1000	301	30.10 ± 0.61 ^c^	30.1	299	29.90 ± 0.39 ^c^	29.9
CIS + **1** 10 mg/kg	1000	311	31.10 ± 0.55 ^c^	31.1	296	29.60 ± 0.47 ^c^	29.6
CIS + **1** 20 mg/kg	1000	305	30.50 ± 0.56 ^c^	30.5	291	29.10 ± 0.66 ^c^	29.1

Negative control—DMSO 1%; cyclophosphamide—100 mg/kg b.w., i.p.; cisplatin—6 mg/kg, b.w., ip. **1**-**1** at doses 5, 10, 20 mg/kg b.w., i.p. CYP + **1** cyclophosphamide—100 mg/kg (b.w.; i.p.) + **1** at doses 5, 10, 20 mg/kg (b.w., i.p.); CIS + **1**-cisplatin—6 mg/kg (p.c.; i.p.) + **1** at doses 5, 10, 20 mg/kg (b.w., i.p.). Legend: A.V.—Absolute value. Different letters indicate statistically significant differences. Statistical test: ANOVA/Tukey.

**Table 6:** Ligand–receptor complex interaction energies with B-DNA (crystallized).

Compound	Docking Score (kcal/mol)
**1**	−88.75
cyclophosphamide	−90.39
cisplatin	−69.81

## Data Availability

Not applicable.

## Abbreviation

CIS	cisplatin
CP	cyclophosphamide
DNA	deoxyribonucleic acid
DMSO	dimethyl sulfoxide
RNA	ribonucleic acid
PDB	protein data bank
